# MitoNEET in Perivascular Adipose Tissue Blunts Atherosclerosis under Mild Cold Condition in Mice

**DOI:** 10.3389/fphys.2017.01032

**Published:** 2017-12-19

**Authors:** Wenhao Xiong, Xiangjie Zhao, Minerva T. Garcia-Barrio, Jifeng Zhang, Jiandie Lin, Y. Eugene Chen, Zhisheng Jiang, Lin Chang

**Affiliations:** ^1^Key Laboratory for Atherosclerology of Hunan Province, Institute of Cardiovascular Disease, University of South China, Hengyang, China; ^2^Cardiovascular Research Center, University of Michigan, Ann Arbor, MI, United States; ^3^Life Science Institute, University of Michigan, Ann Arbor, MI, United States; ^4^Department of Cardiac Surgery, University of Michigan, Ann Arbor, MI, United States

**Keywords:** Cisd1, mitoNEET, perivascular adipose tissue, atherosclerosis, mitochondria

## Abstract

**Background:** Perivascular adipose tissue (PVAT), which surrounds most vessels, is de facto a distinct functional vascular layer actively contributing to vascular function and dysfunction. PVAT contributes to aortic remodeling by producing and releasing a large number of undetermined or less characterized factors that could target endothelial cells and vascular smooth muscle cells, and herein contribute to the maintenance of vessel homeostasis. Loss of PVAT in mice enhances atherosclerosis, but a causal relationship between PVAT and atherosclerosis and the possible underlying mechanisms remain to be addressed. The CDGSH iron sulfur domain 1 protein (referred to as mitoNEET), a mitochondrial outer membrane protein, regulates oxidative capacity and adipose tissue browning. The roles of mitoNEET in PVAT, especially in the development of atherosclerosis, are unknown.

**Methods:** The brown adipocyte-specific mitoNEET transgenic mice were subjected to cold environmental stimulus. The metabolic rates and PVAT-dependent thermogenesis were investigated. Additionally, the brown adipocyte-specific mitoNEET transgenic mice were cross-bred with ApoE knockout mice. The ensuing mice were subsequently subjected to cold environmental stimulus and high cholesterol diet challenge for 3 months. The development of atherosclerosis was investigated.

**Results:** Our data show that mitoNEET mRNA was downregulated in PVAT of both peroxisome proliferator-activated receptor gamma coactivator 1-alpha (Pgc1α)- and beta (Pgc1β)-knockout mice which are sensitive to cold. MitoNEET expression was higher in PVAT of wild type mice and increased upon cold stimulus. Transgenic mice with overexpression of mitoNEET in PVAT were cold resistant, and showed increased expression of thermogenic genes. ApoE knockout mice with mitoNEET overexpression in PVAT showed significant downregulation of inflammatory genes and showed reduced atherosclerosis development upon high fat diet feeding when kept in a 16°C environment.

**Conclusion:** mitoNEET in PVAT is associated with PVAT-dependent thermogenesis and prevents atherosclerosis development. The results of this study provide new insights on PVAT and mitoNEET biology and atherosclerosis in cardiovascular diseases.

## Introduction

The major adipose tissue in humans is white adipose tissue (WAT). Although long believed that brown adipose tissue (BAT) only existed in infants, the existence of a functional BAT is now accepted in the clavicular, cervical, suprarenal, and periaortic regions of adult humans (Nedergaard et al., 2007, 2010). WAT and BAT exhibit distinct functions. WAT has been recognized as a tissue for energy storage (Kim and Moustaid-Moussa, 2000) and related to cardiovascular diseases (CVDs), while the main function of BAT is to generate heat and energy expenditure (Smith and Horwitz, 1969). Studies using mouse models demonstrated that activation of BAT by cold temperature enhances clearance of plasma lipids and prevents the development of atherosclerosis (Bartelt et al., 2011). Additionally, WAT can be converted to “beige” fat (between WAT and BAT) by cold stimuli or hormones such as irisin (Zhang et al., 2014), FGF21 (Fisher et al., 2012), etc. Also, beige can be converted to WAT (Cohen et al., 2014). Beige adipocytes have a gene expression pattern distinct from either WAT or BAT and promote energy expenditure due to existence of uncoupling protein-1 (UCP-1) in the mitochondria, similarly to classic brown adipocytes (Wu et al., 2012). Recent strategies for “browning” WAT via cold stimuli or hormones significantly enhanced thermogenesis and may aid in prevention of obesity and related CVDs. Additionally, not all adipose tissue expansion is necessarily associated with pathological changes. The concept of the “metabolically healthy obese” state (Ruderman et al., 1981) suggests that some individuals can preserve systemic insulin sensitivity on the basis of “healthy” adipose tissue expansion (Sun et al., 2011). One example is that of thiazolidinediones (TZDs), the insulin-sensitizers known to affect the morphology of adipose tissue while improving insulin sensitivity. Both in humans and experimental animals, TZDs increase the number of small adipocytes and decrease large adipocytes (Hallakou et al., 1997; Okuno et al., 1998; Boden et al., 2003). TZDs have favorable effects on atherosclerosis in patients with type 2 diabetes mellitus (Ryan et al., 2007; Yu et al., 2007; Harashima et al., 2009; Kiyici et al., 2009).

PVAT is the adipose tissue surrounding most vessels. The PVAT of rodents is similar to BAT (Chang et al., 2012b). We previously demonstrated that one major physiological function of PVAT is thermogenesis in response to cold stimuli (Chang et al., 2012b). The intrinsic characteristic of energy expenditure of brown or beige adipocytes highlights the potential importance of PVAT as a target for the treatment of obesity and related CVDs (Chang et al., 2017). Our previous study demonstrated that donor PVAT from healthy mice ameliorates the endothelial dysfunction of aging recipient mice (Chang et al., 2012b) and that cold exposure inhibits atherosclerosis and improves endothelial function in mice with intact PVAT but not in mice lacking PVAT. Thus, our published data strongly suggest that PVAT metabolism is highly related to atherosclerosis.

MitoNEET, a mitochondrial outer membrane protein necessary for energy metabolism, was identified as an additional target of TZDs (Colca et al., 2004; Wiley et al., 2007a,b). Adipocyte specific overexpression of mitoNEET driven by the aP2 promoter induces severe obesity in ob/ob mice. Surprisingly, the adipocyte size was normal in the mitoNEET transgenic ob/ob mice despite accumulation of fat mass, suggesting that mitoNEET is able to convert hypertrophic fat to hyperplastic fat in ob/ob mice (Kusminski et al., 2012). Our study shows that mitoNEET expression is dramatically reduced in PVAT of *Pgc1*α or *Pgc1*β knockout mice which exhibit impaired PVAT thermogenesis. Thus, we hypothesize that mitoNEET is a critical mediator to maintain PVAT thermogenesis and protects against atherosclerosis. In this study, we document that the mice with specific overexpression of mitoNEET in brown adipocytes (mitoNEET-Tg) are cold resistant and partially resistant to the development of atherosclerosis in an ApoE knockout background.

## Materials and methods

### Animals

Transgenic mice with brown adipocyte-specific overexpression of mitoNEET (mitoNEET-Tg) in a C57BL/6J background were generated to express human mitoNEET driven by the mouse *Ucp-1* promoter. Littermate mice without the human mitoNEET transgene served as wild type control mice. For the atherosclerosis study, mitoNEET-Tg mice were crossed with ApoE knockout (ApoE KO) mice (Stock# 002052, Jackson Laboratory) to obtain ApoE knockout mitoNEET-Tg mice (ApoE/mitoNEET-Tg). The offspring were genotyped by PCR analysis of DNA obtained from tail-snip biopsies using transgene-specific oligonucleotide primers for human mitoNEET and ApoE knockout. We selected two groups of mice for this study: (1) ApoE homozygous and positive for human mitoNEET, and (2) littermate control mice with a genotype of ApoE homozygous and negative for human mitoNEET. All experiments were conducted in 8-week-old male mice. The study protocol was approved by the Animal Research Ethics Committee of the University of Michigan.

### Surgical removal of the interscapular BAT

Mice were anesthetized by isoflurane inhalation and fixed face down on a surgical heating pad (37°C). The subscapular hair was removed, and a 1 cm long incision on the midline skin was made to expose the interscapular BAT. Next, the intact BAT was completely separated from the interscapular trigonal pyramidal region. The vessels supplying blood to BAT and the neighboring cells at the trigonal pyramidal bottom were cauterized using an electronic cauterizer to permanently block bleeding and BAT regeneration after the BAT removal procedure. The skin wound was closed using wound clips. The mice were allowed to recover for 1 week at room temperature (22°C) before initiating the temperature, energy expenditure and atherosclerosis studies at 16°C (Chang et al., 2012b).

### Wireless measurements of body temperature using implanted probes

The mice were anesthetized by isoflurane inhalation. The neck hair was removed, and the skin was opened. A temperature monitoring microchip (Bio Medic Data Systems, 12 mm long and 2 mm in diameter) was surgically implanted in the subcutaneous area. After 3 days' recovery from the surgery, the temperature was manually recorded by remotely scanning the animal using a handheld reader system (DAS-7007R, Bio Medic Data Systems) at 9 a.m., 12 p.m., and 4 p.m. daily.

### Measurement of intravascular temperature in mice

Intravascular temperature was monitored using a T-type thermocouple probe (ADInstruments MLT1405) which was inserted into the thoracic aorta through the left carotid artery of mice under anesthesia induced by isoflurane inhalation (Chang et al., 2012b). The thermocouple probe was connected to a data acquisition system (AdInstruments Powerlab) to monitor the temperature inside of aortic lumen. During the procedure, the mice were lying on their backs on a 35°C warm pad with constant inhalation of isoflurane, and cold stimulation was performed by submerging the tail and hind feet in 4°C water with the researcher blinded to the genotype of mice.

### Energy expenditure assay in mice

Oxygen consumption (VO_2_), carbon dioxide production (VCO_2_), spontaneous motor activity and food intake were measured using the Comprehensive Laboratory Monitoring System (CLAMS, Columbus Instruments), an integrated open-circuit calorimeter equipped with an optical beam activity monitoring device. Mice were individually placed into the sealed chambers (7.9′′ × 4′′ × 5′′) with free access to food and water. To determine the energy expenditure in mice upon acute cold exposure, the study was carried out in an experimentation room set at 22 or 4°C with 12-12 h (6:00 p.m.~6:00 a.m.) dark-light cycles. The measurements were carried out continuously for 48 h at 22 or 4°C. Mice were provided food and water through the feeding and drinking devices located inside the chamber without nesting material due to the fact that it blocks the beams that track activity. The amount of food consumed by each animal was monitored through a precision balance attached below the chamber. The body composition data were measured using an NMR analyzer when conscious mice were placed individually into the measuring tube with a minimum restrain. Total energy expenditure was calculated based on the values of VO_2_, VCO_2_, and the protein breakdown (Riachi et al., 2004).

### Atherosclerosis study

For atherosclerosis experiments, 8-week-old male ApoE KO and ApoE/mitoNEET-Tg mice were fed a high-cholesterol diet (Harlan, TD.88137) for 3 months in a cold-temperature chamber (16°C) with a 12-h:12-h light-dark cycle and free access to water and diet as in our previous study (Chang et al., 2012b). Afterwards, the animals were sacrificed with excess CO_2_. After collection of plasma, the mice were perfused with 20 ml normal saline solution through the heart, followed by 20 ml 37% formalin. The mice were fixed with formalin and the whole aortic tree was dissected under a surgical microscope. Next, the aortic trees were stained with Oil Red O solution (0.2% Oil Red O (w/v) in 3.5:1 of methanol:1N NaOH) for 50 min, followed by 70% ethanol for 30 min. Afterwards, the aortic trees were kept in ddH_2_O. The attached connective tissues around the aortic trees were cleaned and pinned on a plate containing paraffin wax, and then the aorta was longitudinally opened with a Vannas scissor to expose the atherosclerotic lesions. The pictures of whole aortic trees were obtained using a digital camera, and the atherosclerotic lesion areas were calculated by an Image software (Meta Imaging Series 7.0, Molecular Devices, LLC).

### Histological analysis

Adipose tissues were harvested from mice that were anesthetized and fixed overnight via transcardial perfusion with 4% paraformaldehyde (pH 8.0). After dehydration, the samples were embedded in paraffin wax according to standard laboratory procedures. Sections of 5 μm were stained with H&E for routine histopathological examination with light microscopy.

### Quantitative real-time reverse-transcriptase polymerase chain reaction (QT-PCR) and western blot

The mice were housed at 4°C for 24 h, 16°C for 1 week or 3 months. The tissues indicated in the figures were harvested and frozen in liquid nitrogen for mRNA and protein analysis. Total RNA was isolated from tissues using TRIzol reagent (Invitrogen). The mRNA levels were measured by QT-PCR using a Bio-Rad thermocycler and a SYBR green kit (Bio-Rad). The mouse primers used for each gene were, respectively, as follows:

*Ucp-1*: 5′-AAAAACAGAAGGATTGCCGAAACT-3′ and 5′-TAAGCATTGTAGGTCCCCGTGTAG-3′;

*Cidea:* 5′-CTGTCGCCAAGGTCGGGTCAAG-3′ and 5′-CGAAAAGGGCGAGCTGGATGTAT-3′;

*Pgc-1α:* 5′-CTCCTCCCACAACTCCTCCTCATA-3′ and 5′-GGGCCGTTTAGTCTTCCTTTCCTC-3′;

*Pgc-1β:* 5′-CTACCGCCTGGCCATACCTGTCA-3′ and 5′-CTCCTCATCTTCCTCCCGCTTTTG-3′;

*IL-6:* 5′-TTCCCTACTTCACAAGTC-3′ and 5′-GTACAAAGCTCATGGAGA-3′;

*TNF-α:* 5′-CTCAGATCATCTTCTCAA-3′ and 5′-GGTTTGCCGAGTAGATCT-3′;

*Mcp-1:* 5′-CACCAGCACCAGCCAACTCTCACT-3′ and 5′-CATTCCTTCTTGGGGTCAGCACAG-3′;

*Ckb*: 5′-CCTGCTTCGTCCGGCATC-3′ and 5′-GTCCAAAGTAAAGCCGCTCG-3′;

*Macrod1*: 5′-ATTGTCAACGCTGCCAACAG-3′ and 5′-TTCTGTAGGGTGCGGCATTC-3′;

*Fabp3*: 5′-CAGGTGGCTAGCATGACCAA-3′ and 5′-ATGAGTTTGCCTCCGTCCAG-3′;

*Idh2*: 5′-TGTATCCATGGCCTCAGCAA-3′ and 5′-TGCCATGTACAGAGTACCCAC-3′;

*Klf2*: 5′-TAAAGGCGCATCTGCGTACA-3′ and 5′-GTGGCACTGAAAGGGTCTGT-3′.

The proteins in tissues were extracted by T-PER tissue protein extraction reagent (Thermo Scientific 78510) as indicated in the instructions manual. Protein separation by electrophoresis using 10% SDS-PAGE gels for 1 h at 150V in Tris/Glycine/SDS electrophoresis buffer (25 mM Tris, 190 mM glycine and 0.1% SDS) was performed on 30 μg protein/well in loading buffer (4% SDS, 10% 2-mercaptoethanol, 20% glycerol, 0.004% bromophenol blue and 0.125M Tris-HCl) after boiling at 100°C for 10 min. Proteins were transferred to a nitrocellulose membrane in transfer buffer (25 mM Tris, 190 mM glycine and 20% methanol) at 40V overnight at 4°C. The membrane was rinsed in TBST buffer (20 mM Tris pH7.5, 150 mM NaCl, 0.1% Tween 20) 3 times at room temperature. MitoNEET protein levels were detected by overnight incubation at 4°C in 5% milk containing 1:1000 anti-mitoNEET antibody (ProteintechTM Cat#: 16006-1-AP). Then the membrane was rinsed in TBST buffer, and incubated with Goat anti-Rabbit IgG antibody (IRDye680LT) for 2 h at room temperature. The blot image was captured and analyzed by BioRad LI-COR system.

### Statistical analysis

All data were evaluated with a 2-tailed, unpaired Student's *t*-test or compared by one-way ANOVA followed by Newman-Keuls and were expressed as mean ± SD. A value of *p* < 0.05 was considered statistically significant.

## Results

### MitoNEET is reduced in PVAT of *Pgc1α and Pgc1β* knockout mice

**Figure 1:**
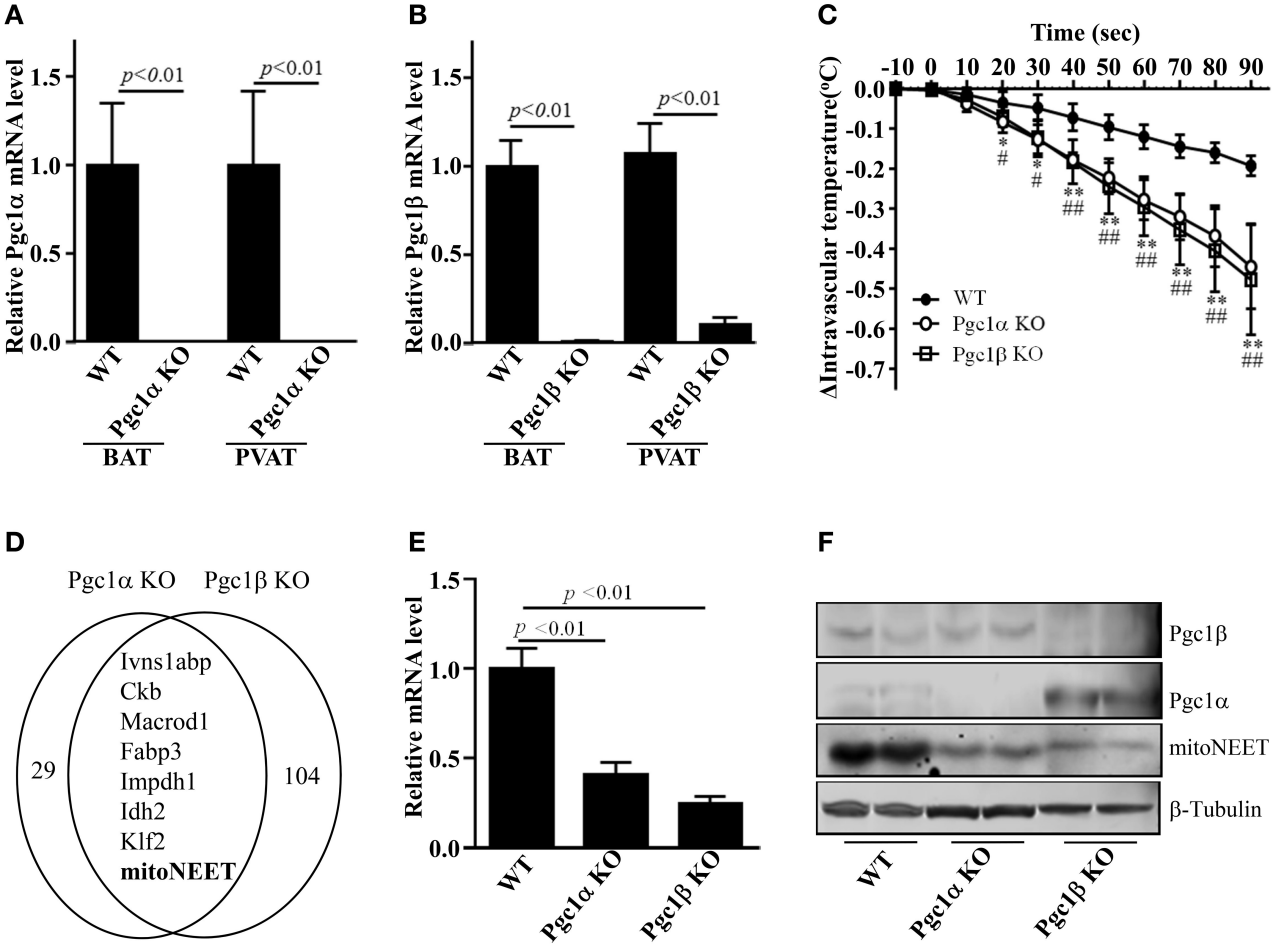
mitoNEET is reduced in PVAT of *Pgc1* knockout mice. Real-time PCR shows *Pgc1*α mRNA levels in BAT and PVAT of *Pgc1*α knockout mice (Pgc1α KO) **(A)**, and *Pgc1*β mRNA levels in BAT and PVAT of *Pgc1*β knockout mice (Pgc1β KO) **(B)**. The relative mRNA levels were normalized by 18S, respectively. Data shown are mean ± SD. *n* = 5 mice/group. **(C)** Intravascular (thoracic aorta) temperature in anesthetized wild type (WT), *Pgc1*α KO and *Pgc1*β KO mice in response to 4°C stimuli. The lag time reflects the time of dipping of the hind feet and tail in 4°C cold water. The zero represents the start time point of immersion in the cold water. Data shown are mean ± SD. *n* = 6 mice/group.^*^p < 0.05 Pgc1α KO vs. WT, ^**^p < 0.01 Pgc1α KO vs. WT; ^#^*p* < 0.05 Pgc1β KO vs. WT, ^##^*p* < 0.01 Pgc1β KO vs. WT. **(D)** RNA deep sequencing identified eight common downregulated genes in PVAT of both *Pgc1*α KO and *Pgc1*β KO mice, as indicated in the Venn diagrams. **(E)** mitoNEET mRNA levels in PVAT of *Pgc1*α KO and *Pgc1*β KO mice. The relative mitoNEET mRNA level was normalized by 18S, respectively. Data shown are mean ± *SD*. *n* = 5 mice/group. **(F)** Western blots show Pgc1α, Pgc1β and mitoNEET protein levels in PVAT of WT, Pgc1α KO and Pgc1β KO mice, *n* = 2 mice/group.

**Figure 2:**
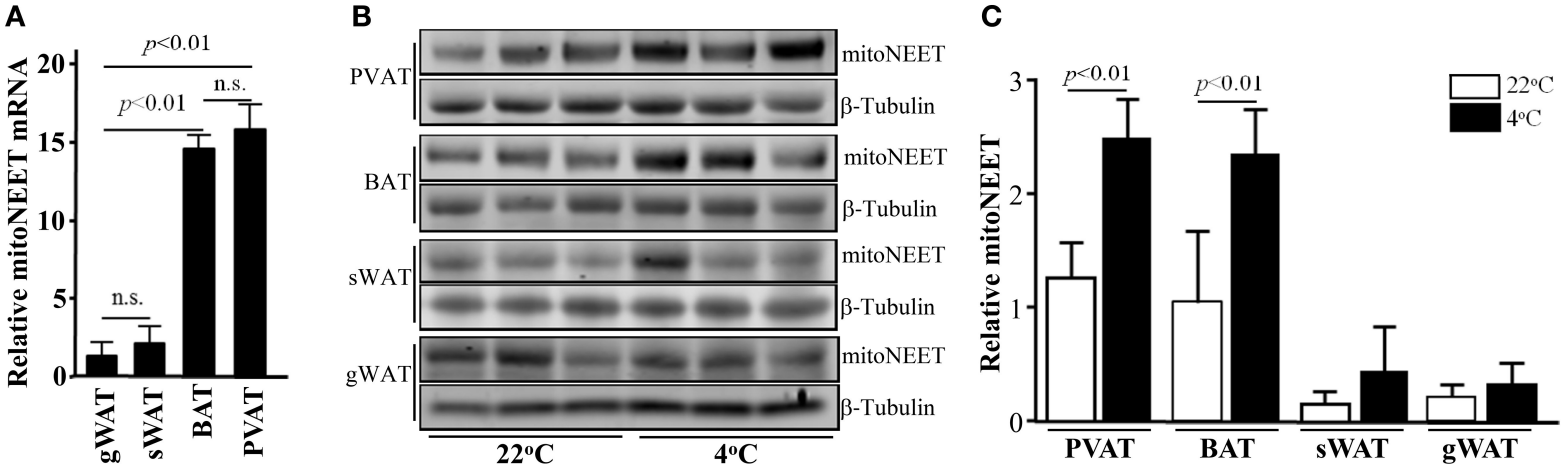
mitoNEET is up-regulated in PVAT upon cold stimuli. **(A)** mitoNEET mRNA levels in BAT, PVAT, gonadal WAT (gWAT), and subcutaneous WAT (sWAT) in 10-week old C57BL/6J mice which were housed at 22°C. The relative mitoNEET mRNA level was normalized by 18S, and the expression level of mitoNEET mRNA in gWAT was set as 1. Data shown are mean ± *SD*. *n* = 5 mice/group. **(B)** Western blots show mitoNEET protein levels in PVAT, BAT, sWAT, and gWAT at 22°C and after 24-h 4°C cold stimuli. *n* = 3 mice per temperature condition. **(C)** Quantitative data of blots in **(B)** expressed as the ratio of densitometry of mitoNEET/β-Tubulin. Data shown as mean ± *SD* of 3 blots either at 22°C or 4°C.

Prior studies indicated that the characteristics of PVAT in mice make it almost identical to interscapular BAT (Fitzgibbons et al., 2011; Chang et al., 2012b). Thus, we reasoned that thermogenesis would be one of the properties of PVAT. Our previous study demonstrated that mice lacking PVAT had lower intravascular temperature in response to cold stimuli (Chang et al., 2012b). Since *Pgc1*α and *Pgc1*β are well-known nuclear receptor coactivators critically involved in BAT thermogenesis (Spiegelman, 2007a,b), we performed RNA deep sequencing analysis in PVAT of *Pgc1*α and *Pgc1*β knockout (KO) mice to screen for factors related to thermogenesis in PVAT. Firstly, we confirmed that *Pgc1*α mRNA or *Pgc1*β mRNA is efficiently deleted in both PVAT and BAT of *Pgc1*α KO (Figure 1A) or *Pgc1*β KO mice (Figure 1B), respectively. To investigate whether *Pgc1*α or *Pgc1*β in PVAT contribute to PVAT thermogenesis, we measured the intravascular temperature of *Pgc1*α or *Pgc1*β KO mice upon cold stimulus for 90 s. As shown in Figure 1C, upon 4°C cold stimulus, the intravascular temperatures in all mice are gradually reduced. However, the intravascular temperature of both *Pgc1*α or *Pgc1*β KO mice drops faster than that of wild type mice. After just 30 s of cold stimulus, the average temperature in thoracic aorta of wild type mice dropped 0.05 ± 0.03°C, while it dropped 0.13 ± 0.04°C and 0.12 ± 0.05°C in the aorta of *Pgc1*α KO and Pgc1β KO mice, respectively. After 60 s of cold stimulus it further dropped to a differential of 0.28 ± 0.05°C and 0.30 ± 0.07°C in *Pgc1*α KO and *Pgc1*β KO mice respectively vs. 0.10 ± 0.03°C in wild type mice. At the endpoint of cold exposure, 90 s, the average temperature in thoracic aorta of wild type mice is reduced 0.19 ± 0.03°C while it is reduced 0.44 ± 0.11°C in *Pgc1*α KO mice and 0.48 ± 0.14°C in *Pgc1*β KO mice (Figure 1C). These data indicate that deleting *Pgc1*α or *Pgc1*β in PVAT might contribute to the hypothermic phenotype independent of BAT. To investigate the common thermogenic genes, which might represent a cross point between *Pgc1*α and *Pgc1*β in terms of their shared thermogenic mechanisms, we compared the genes in PVAT of *Pgc1*α and *Pgc1*β showing a 1.5-fold change when compared to PVAT of wild type mice. About 37 genes are reduced in PVAT of *Pgc1*α KO mice, while about 112 genes are reduced in PVAT of *Pgc1*β KO mice. Among them, only 8 genes [*Ivns1abp, Ckb, Macrod1, Fabp3, Lmpdh1, Idh2, Klf2* and *Cisd1* (mitoNEET)], which might be directly involved as effectors of thermogenic mechanisms, are reduced in PVAT of both *Pgc1*α and *Pgc1*β KO mice (Figure 1D), consistent with known functions of these genes in thermogenesis and lipid metabolism in adipose tissue (Banerjee et al., 2003; Chen et al., 2011; Vergnes et al., 2011; Van der Zee, 2013; Lee et al., 2016). Using real-time PCR, we confirmed that 6 of 8 genes were down-regulated in PVAT of both *Pgc1*α and *Pgc1*β KO mice (Suppl. Figure IA, Figure 1E). MitoNEET protein levels were also significantly reduced in PVAT of *Pgc1*α and *Pgc1*β KO mice (Figure 1F). Although all the common differentially regulated genes might be critical for thermogenesis, we focused on mitoNEET in this study because mitoNEET is located on the mitochondrial membrane, making it a likely direct effector, and it is involved in WAT browning (Kusminski et al., 2014). Also, compared with WAT, mitoNEET mRNA levels in PVAT and BAT are about 15-fold higher, respectively (Figure 2A). These data suggest that mitoNEET in brown-like PVAT might be directly involved in the regulation of PVAT-dependent thermogenesis.

### MitoNEET is up-regulated in brown-like adipocytes upon cold stimulus

Next, we investigated whether mitoNEET is associated with thermogenesis. Even though mitoNEET is also highly expressed in mitochondria-rich organs such as skeletal muscle, heart and brain, however, upon cold stimulus, mitoNEET expression is not increased in those three tissues (Suppl. Figure I) while it is significantly increased in PVAT and BAT (Figures 2B,C). Despite of subcutaneous WAT (sWAT) being recognized as an Ucp1-positive beige adipose tissue, the mitoNEET mRNA in sWAT is comparable with that in pure gonadal WAT (gWAT) (Figure 2A). Consistently, the increase in mitoNEET levels in sWAT is not as marked as in PVAT and BAT (Figures 2B,C) upon 24 h cold stimulus. These data suggested that mitoNEET in brown-like PVAT might be strongly associated with cold-induced thermogenesis.

### Mice with MitoNEET overexpression in brown adipocytes are cold resistant

**Figure 3:**
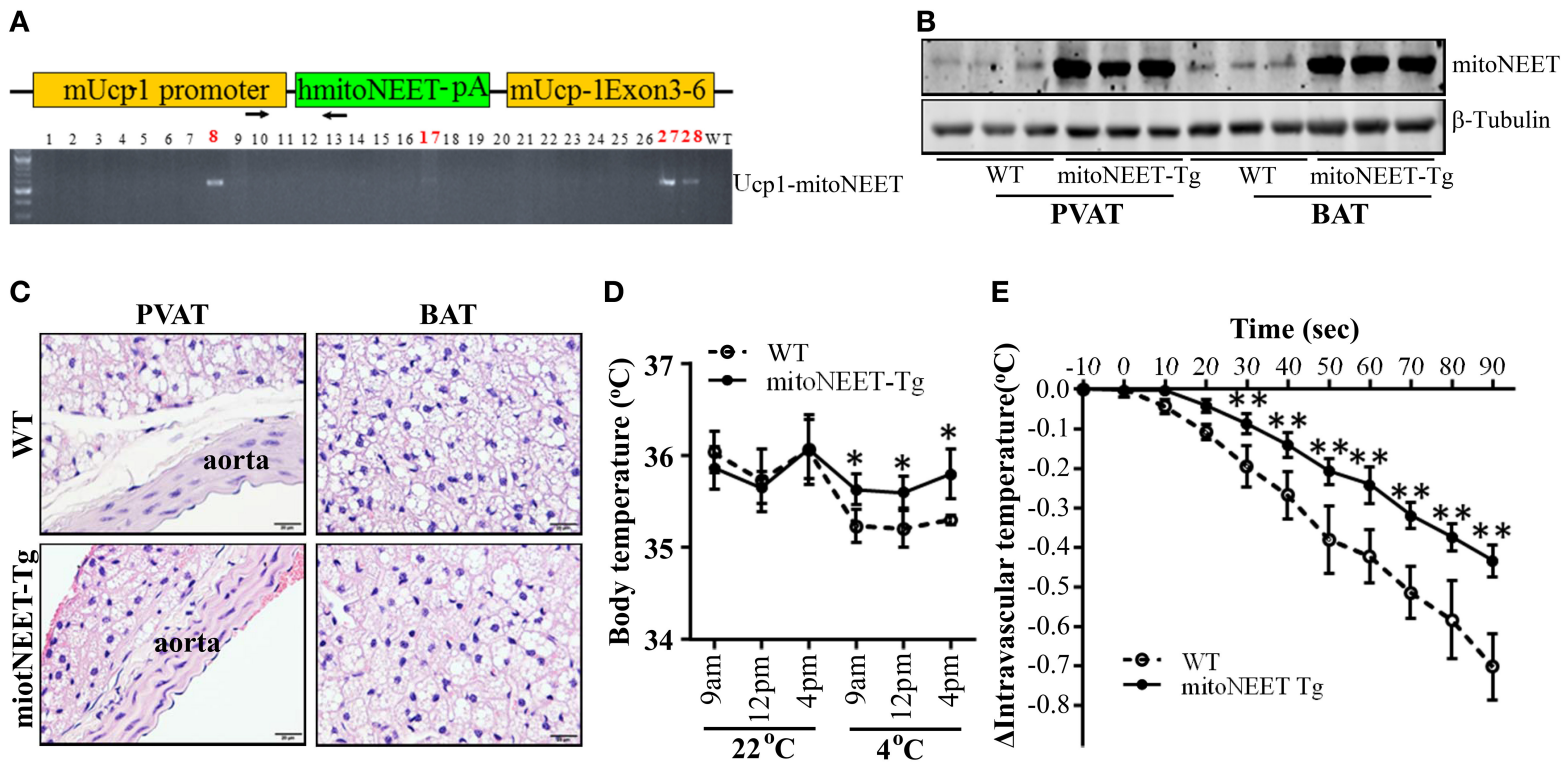
Cold tolerance in mitoNEET-Tg mice**. (A)** Schema of the construct used for generating transgenic mice with brown adipocyte-specific overexpression of the human mitoNEET driven by the mouse Ucp-1 promoter (top). Identification of four human mitoNEET positive founders in the C57BL/6J background (#8, #17, #27, and #28) were identified (bottom). The transgenic mice used in this study are from #8 line. **(B)** Western blot shows that mitoNEET is overexpressed in PVAT and BAT of the transgenic mice. **(C)** Representative H.E. staining showing the morphology of thoracic aortic PVAT and interscapular BAT in 10-week old wild type and mitoNEET-Tg mice. Magnification bar = 20 μm. **(D)** Body temperature of conscious wild type and mitoNEET-Tg mice in response to 4°C stimuli. The body temperatures were collected at 9 a.m., 12 p.m., and 4 p.m. when the mice were housed either in a 22°C or a 4°C chamber. Data shown as mean ± SD, *n* = 5 mice per group,^*^p < 0.05 vs. WT mice. **(E)** Intravascular (thoracic aorta) temperature in the anesthetized mice with interscapular BAT removal was recorded for 90 s as described in Materials and Methods Section. Data shown as mean ± SD. *n* = 6 mice/group.^**^p < 0.01 vs. WT.

**Figure 4:**
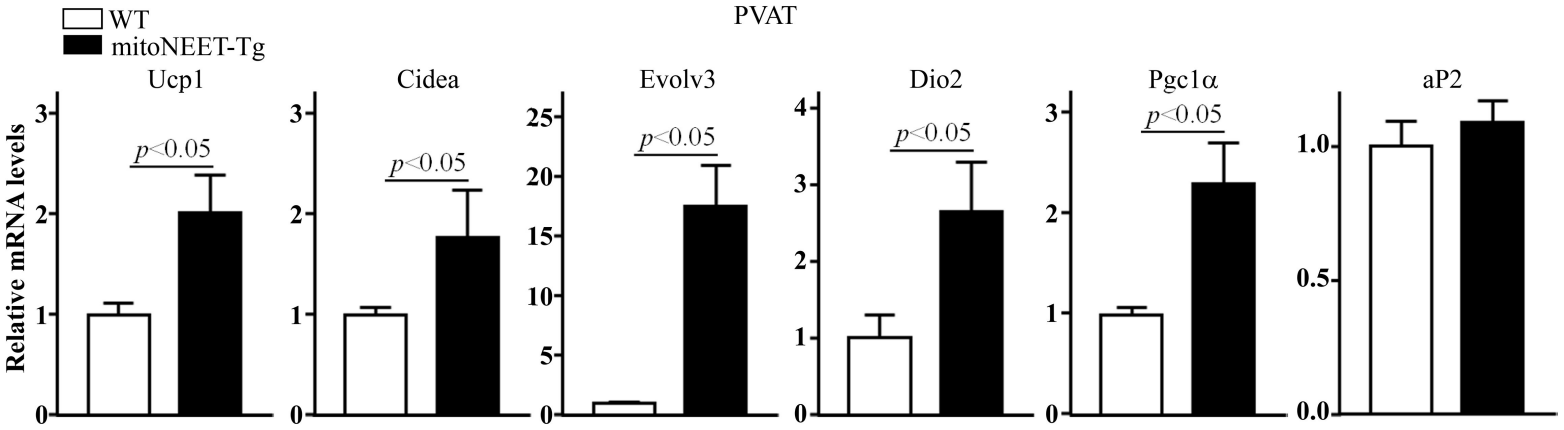
Increase in thermogenesis-related genes in PVAT of mitoNEET-Tg mice. RT-PCR was used to determine the mRNA levels (relative to 18S) of thermogenesis-related genes in PVAT of wild type and mitoNEET-Tg mice housed at 16°C for 1-week. Data shown as mean ± SD. *n* = 6 mice/group.

To investigate whether mitoNEET in brown adipocytes regulates thermogenesis, we generated mice with brown adipocyte-specific overexpression of human mitoNEET (mitoNEET-Tg) driven by the *Ucp-1* promoter using the strategy outlined in Figure 3A. Western blot confirms that mitoNEET is specifically overexpressed in PVAT and BAT (Figure 3B), but not in subcutaneous and gonadal WAT (Suppl. Figure IIA). Overexpression of mitoNEET in brown adipocytes does not affect the morphology of interscapular BAT, PVAT and aorta (Figure 3C), or of subcutaneous and gonadal WAT (Suppl. Figure IIB). Next, we investigated whether mitoNEET overexpression in brown adipocytes enhances thermogenesis in mice. As shown in Figure 3D, in a 22°C environment, the body temperatures are comparable between wild type and mitoNEET-Tg mice. However, when the mice are placed in a 4°C environment, the body temperature is significantly reduced in the wild type animals while the mitoNEET-Tg mice are able to maintain the body temperature, suggesting that mitoNEET in brown adipocytes is able to prevent the temperature reduction observed in the wild type animals upon transfer to the cold environment. To exclude the potential contribution of BAT to intravascular temperature, we surgically removed the interscapular BAT in both the wild type and mitoNEET-Tg mice and measured the intravascular temperature in mice under anesthesia. As shown in Figure 3E, by immersing the hind feet and tail of mice with 4°C cold water, the reduction rate of intravascular temperatures of mitoNEET-Tg mice is slower than that of wild type mice. After 90 s of cold stimulation, the intravascular temperature in wild type mice dropped by 0.70 ± 0.08°C, while it only dropped by 0.44 ± 0.04°C in mitoNEET-Tg mice. Consistently, in mice housed in a mild cold environment (16°C) as a challenge for 1-week, the mRNA levels related to thermogenesis-associated genes such as *Ucp1, Cidea, Evolv3, Dio2*, and *Pgc1*α are increased in the PVAT of mitoNEET-Tg mice when compared with those in PVAT of wild type mice (Figure 4) while the adipogenic *aP2* remains unchanged. These genes, except for *Evolv3* are not increased in gWAT (Suppl. Figure IIIA). The increased expression of thermogenesis-associated genes in PVAT was also confirmed in the mitoNEET-Tg mice line originated from founder #27 (Suppl. Figure IIIB), ruling out an insertional effect in the #8 founder. These data further confirm that mitoNEET in PVAT positively contributes to cold-induced thermogenesis independently of the presence of BAT.

### Overexpression of MitoNEET in PVAT increases whole body metabolism

**Figure 5:**
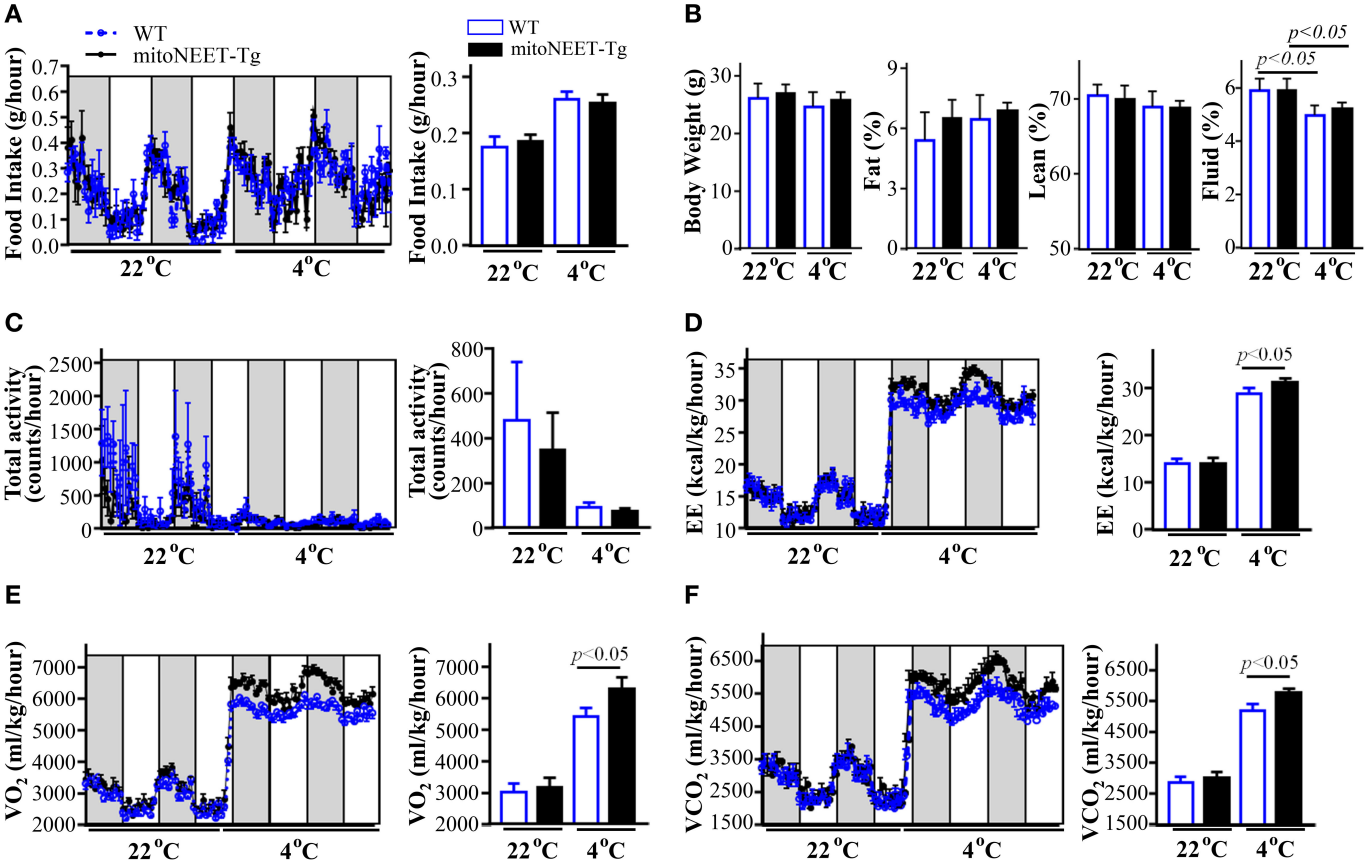
Energy expenditure in mitoNEET-Tg mice. Wild type and mitoNEET-Tg mice with interscapular BAT removed were single-housed in metabolic cages. The food intake **(A)**, body weight and body composition **(B)**, total locomotor activity **(C)**, energy expenditure **(D)**, oxygen consumption **(E)** and carbon dioxide production **(F)** were recorded when the chamber temperatures were adjusted to 22°C or 4°C. Data shown as mean ± S.E.M (*n* = 6) and histogram figures are the 24-h average.

Next, we studied whether overexpression of mitoNEET in PVAT from mice with BAT removed promotes energy expenditure. Our results indicate that overexpression of mitoNEET in brown adipocytes does not affect the food intake (Figure 5A), body composition (Figure 5B) and total locomotor activity (Figure 5C) when the mice were housed in a 22°C environment. Additionally, there are no statistical differences in energy expenditure at room temperature, between that of wild type and mitoNEET-Tg mice (Figure 5D). Consistently, O_2_ consumption (Figure 5E) and CO_2_ production (Figure 5F) are comparable between wild type and mitoNEET-Tg mice at that temperature. When mice were housed at 4°C, the cold stimulus increases comparably the food intake and reduces fluid percentage and total locomotor activity of both wild type and mitoNEET-Tg mice (Figures 5A–C), indicating that overexpression of mitoNEET in PVAT does not change the food intake, body composition and movement behaviors of mice. However, when challenged with 4°C cold stimulus, both O_2_ consumption (Figure 5E) and CO_2_ production (Figure 5F) are significantly increased in the mitoNEET-Tg mice. Consistently, the energy expenditure (Figure 5D) was also significantly increased at 4°C in mitoNEET-Tg mice when compared with wild type mice, suggesting that mitoNEET in PVAT is involved in cold-induced energy expenditure.

### Overexpression of MitoNEET in PVAT attenuates the development of atherosclerosis in mice

**Figure 6:**
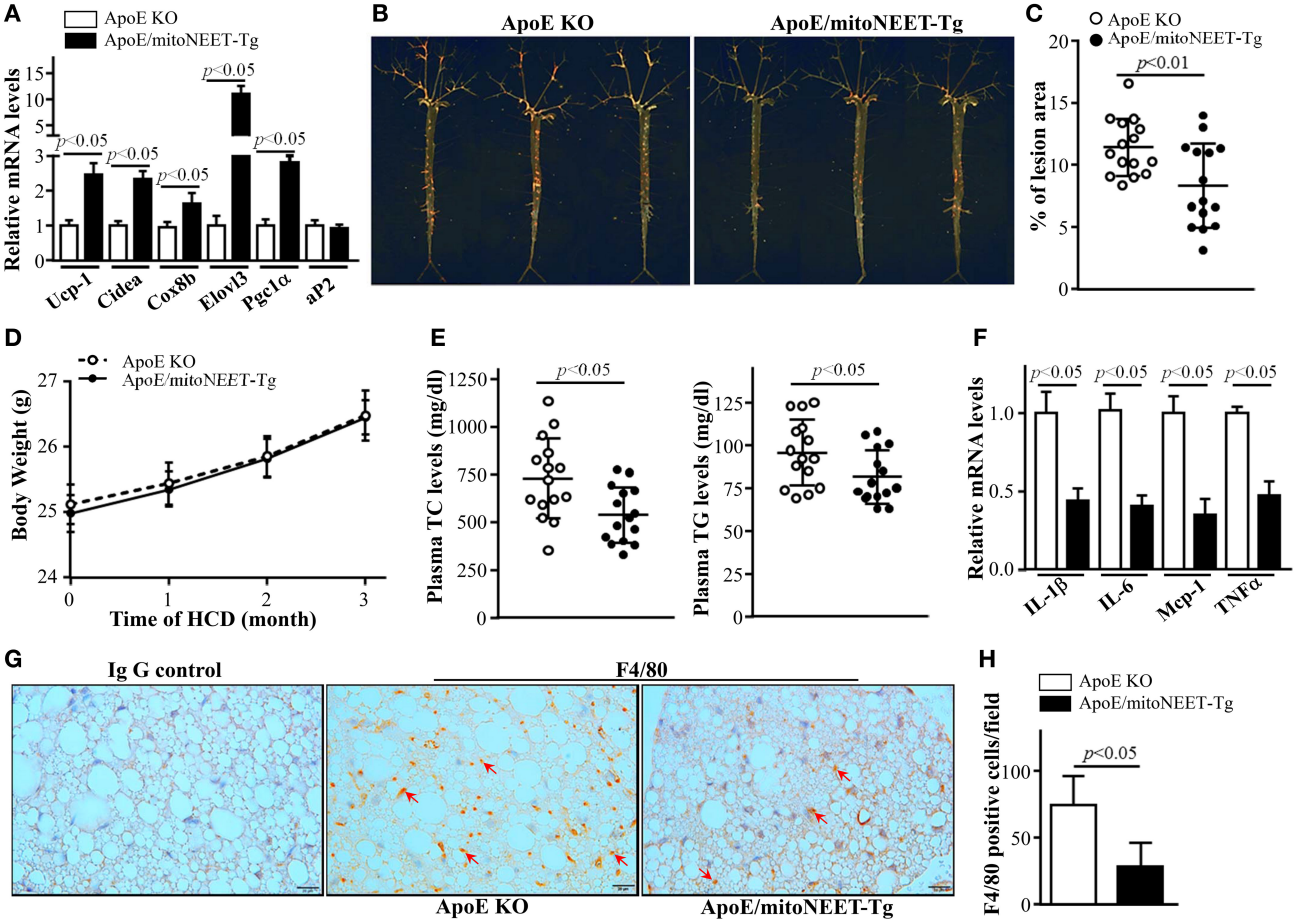
Atherosclerosis in mitoNEET-Tg mice. Twelve-week-old ApoE KO and ApoE/mitoNEET-Tg mice were housed at and fed a high cholesterol diet (HCD) at 16°C for 3 months. **(A)** mRNA levels of thermogenesis genes in PVAT of mice at the end-point of the atherosclerosis study. Data shown are mean ± S.E.M. *n* = 5–6 in each group. **(B)** Representative Oil Red O staining showing atherosclerotic lesions in whole aortic trees. **(C)** Quantitative analysis of the ratio of atherosclerotic lesion area to total aortic tree area. Data shown are mean ± SD. *n* = 15 in each group. **(D)** Body weights of mice during mild cold challenge. Data shown are mean ± S.E.M. *n* = 15 in each group. **(E)** Plasma total cholesterol and triglyceride levels in ApoE knockout and ApoE/mitoNEET-Tg mice at the end-point of the HCD challenge at 16°C for 3 months. Data shown are mean ± SD. *n* = 15 in each group. **(F)** mRNA levels of inflammatory genes in PVAT of mice at the end-point of HCD challenge at 16°C for 3 months. Data shown are mean ± S.E.M. *n* = 5–6 in each group. **(G)** Macrophage infiltration detected by F4/80 staining (brown) in PVAT of mice at the end-point of HCD challenge at 16°C for 3 months. **(H)** Quantitative data of F4/80 positive cells in **(G)**. Data shown are mean ± SD. *n* = 6 in each group.

Since acute 4°C cold exposure enhanced thermogenesis and energy expenditure in mitoNEET-Tg mice, we investigated the effects of overexpression of mitoNEET in PVAT on atherosclerosis when the interscapular BAT was removed and the mice were housed in a mild cold 16°C environment for 3 months. As shown in Figure 6A, after 16°C exposure and high cholesterol diet feeding for 3 months, ApoE/mitoNEET-Tg mice show higher expression of thermogenesis-related genes such as *Ucp-1, Cidea, Cox8b, Evolv3*, and *Pgc1a* in PVAT than those in the PVAT of ApoE knockout mice. Consistently, *en face* staining of lipid-rich lesions from mouse aortas showed that the total lesion area was significantly lower in aortas from ApoE/mitoNEET-Tg than those from ApoE knockout mice (Figures 6B,C), indicating that overexpression of mitoNEET in PVAT inhibits atherosclerosis in mild cold conditions. However, the body weights (Figure 6D) are comparable between ApoE knockout mice with and without mitoNEET overexpression in PVAT after 16°C exposure for 3 months. Compared to ApoE knockout mice, the total plasma cholesterol and triglyceride levels in ApoE/mitoNEET-Tg mice are significantly reduced in mild cold conditions (Figure 6E), suggesting increased lipid clearance. Remarkably, overexpression of mitoNEET significantly reduced the expression of the pro-inflammatory genes *IL-1*β*, IL-6, Mcp-1*, and *TNF*α in PVAT of ApoE/mitoNEET-Tg mice (Figure 6F). Consistently, the macrophage infiltration is significantly reduced in PVAT of ApoE/mitoNEET-Tg mice (Figures 6G,H). Taken together, these data indicate that increased levels of mitoNEET in PVAT reduce the development of atherosclerosis.

## Discussion

Decreasing the environmental temperature initiates BAT-adaptive thermogenesis in mammals due to uncoupled ATP generation by *Ucp1* in the mitochondria and dissipates energy in the form of heat (Enerback et al., 1997; Lee et al., 2014). Because of their high expression levels in BAT, *Pgc-1* coactivators are well-established markers of BAT. *Pgc1*α and *Pgc1*β are transcriptional coactivators which recruit nuclear receptors or transcription factors to regulate transcription of downstream genes in the nucleus and the mitochondria (Kadlec et al., 2016), and play an absolutely essential and complementary function in mitochondrial biogenesis and thermogenesis in BAT (Puigserver et al., 1998; Lelliott et al., 2006; Uldry et al., 2006). Therefore, we compared the common genes in PVAT found down-regulated in both *Pgc1*α and *Pgc1*β deficient mice, which might represent key points of commonality in the thermogenesis roles of both *Pgc-1* coactivators. Our study here indicates that mitoNEET is one of the molecules which are highly down-regulated in PVAT in both *Pgc1*α and *Pgc1*β deficient mice. MitoNEET, a dimeric mitochondrial outer membrane protein, is a key regulator of mitochondrial function and lipid homeostasis. Loss of mitoNEET in cells decreases cellular respiration because of reduction in the total cellular mitochondrial volume, suggesting that mitoNEET plays critical roles in controlling mitochondrial homeostasis (Vernay et al., 2017). In adipocytes, mitoNEET reduces β-oxidation rates by inhibiting mitochondrial iron transport into the matrix. Interestingly, adipocyte-specific overexpression of mitoNEET driven by the *aP2* promoter enhances lipid uptake and leads to benign adipose tissue hyperplasia. Despite the severe obesity resulting from mitoNEET overexpression in ob/ob mice, the insulin sensitivity is preserved (Kusminski et al., 2012). Therefore, mitoNEET might be a potential therapeutic target for diabetes. Additionally, beside its positive impact on lipid and carbohydrate homeostasis by altering mitochondrial matrix iron metabolism, mitoNEET in subcutaneous WAT of mice upregulates a browning signature program (Kusminski et al., 2014). Recently, browning of WAT is passionately recognized as a new strategy for treatment of diabetes and CVDs. Importantly, we documented that mitoNEET is highly expressed in brown-like PVAT and BAT when compared to WAT. Cold exposure significantly increases mitoNEET expression in PVAT and BAT, but not in WAT and other mitochondria-rich organs such as skeletal muscle, heart and brain, suggesting that mitoNEET expression is highly and directly associated with thermogenesis in brown adipocytes. These findings are consistent with the possibility that PVAT could undergo heat generation upon cold stimulus and prompted our further characterization of the role of this gene in PVAT.

Impaired energy metabolism in the blood vessels is believed to be associated with atherogenesis (Mayr et al., 2005). Environmental temperature influences the energy metabolism in the body (Balogh et al., 1952). Even though exposure of mice to 4°C enhances thermogenesis, long-term (8 weeks) 4°C exposure promotes atherosclerotic plaque growth and instability due to Ucp1-dependent lipolysis of adipose tissues (Dong et al., 2013). On the other hand, as PVAT has a phenotype similar to BAT (Fitzgibbons et al., 2011; Chang et al., 2012b), the heat generation in PVAT is critical to the maintenance of intravascular temperature (Chang et al., 2012b). In humans, an intravascular temperature gradient exists, with the temperature increasing in large veins (surrounded by PVAT) as blood approaches the heart (Robinson, 1952), although it is not yet known if this function is associated with PVAT. We previously showed that, consistent with a potential activation of PVAT metabolism during cold-induced thermogenesis, the presence of PVAT in ApoE knockout mice on a high-fat diet and housed in a mild cold temperature (16°C) facility prevented atherosclerosis compared to mice housed at room temperature (22°C) (Chang et al., 2012b). PVAT-free mice housed in similar cold conditions did not have comparable reductions in atherosclerosis, underscoring the necessity of PVAT for this phenotype. Yet, the factors contributing to those phenotypes are still unknown. Here we found that overexpression of mitoNEET in PVAT up-regulates expression of thermogenic genes such as *Ucp-1, Cidea, Dio2* and *Pgc1*α, etc. in cold conditions. Consistently, mitoNEET-Tg mice (when the BAT was surgically removed) are cold tolerant, indicating that mitoNEET in PVAT contributes to thermogenesis. Indeed, in our study, we found that mitoNEET-Tg mice housed in 4°C environment increased systemic metabolic activity. Also, housing mitoNEET-Tg mice at 16°C for 3-months significantly reduced the development of atherosclerosis when compared with ApoE knockout mice. Importantly, plasma triglyceride levels in mitoNEET-Tg mice were reduced at 16°C, suggesting that the increased metabolic activity of PVAT in mitoNEET-Tg mice may result in increased lipid clearance from the vasculature, thereby contributing to reduced atherogenesis. Indeed, activation of BAT (Bartelt et al., 2011) and PVAT (Chang et al., 2012a) in rodents results in reduced plasma lipid levels. In humans, studies have reported that individuals living in cold climates have active BAT in the peri-aortic region of adults (van Marken Lichtenbelt et al., 2009). However, it is yet unclear if cold exposure in humans activates PVAT thermogenesis leading to protection from atherosclerosis. Exposure to both heat and cold are associated with increased incidences of mortality from heart attacks in humans (Taggart et al., 1972; Sheldahl et al., 1992) although we still need carefully-controlled epidemiological studies to determine if cold exposure is beneficial in preventing the development of atherosclerosis.

PVAT is closely involved in vascular inflammation. The PVAT-resident and -recruited inflammatory cells have been hypothesized to be responsible for vascular diseases (Okamoto et al., 2001). It is believed that the inflammatory response in the vasculature is a key step toward atherosclerosis. Indeed, high-fat diet feeding induces a pro-inflammatory phenotype in the PVAT of mice (Chatterjee et al., 2009). Actually, compared with subcutaneous and visceral adipose tissues, PVAT has less-differentiated adipocytes with more basal inflammatory signature, and lower expression of adiponectin and higher of inflammatory factors such as *IL-6, IL-8*, and *MCP-1* (Chatterjee et al., 2009). Indeed, accumulation of inflammatory cells in the PVAT in human atherosclerotic aortas indicates that PVAT recruits pro-inflammatory cells in atherogenesis and is primed for inflammatory responses (Henrichot et al., 2005). Transplant of pro-inflammatory visceral WAT results in atherosclerotic lesions and increased inflammatory markers, compared to transplantation of non-inflammatory subcutaneous WAT (Ohman et al., 2008, 2011). A postmortem study also found that the PVAT mass was positively correlated with atherosclerotic plaque size in atherosclerosis patients (Verhagen et al., 2012). Therefore, an inflammatory PVAT plays pro-atherosclerotic roles. Surprisingly, our data uncovered that expression of inflammatory factors such as *IL-6* and *Mcp-1* is reduced in PVAT of mitoNEET-Tg mice, suggesting an anti-inflammatory role for mitoNEET likely contributing further to the atheroprotective phenotype. Indeed, macrophage infiltration was reduced in the mitoNEET-Tg mice.

Taken together, our study demonstrates that mitoNEET in PVAT plays a key role in intravascular thermoregulation. mitoNEET in PVAT prevents temperature loss in the vasculature upon cold temperature challenge. Of great importance, we show that mitoNEET in PVAT reduces the burden of atherosclerosis, making it an attractive target for clinical intervention, and establishes the notion of a direct beneficial impact of mitoNEET in PVAT to reduce cardiovascular diseases.

## Author contributions

WX, XZ, JZ, and LC designed and performed the experiments; JL provided Pgc1α and Pgc1β knockout mice; LC, YC, ZJ, analyzed the data; LC and MG-B wrote the paper.

### Conflict of interest statement

The authors declare that the research was conducted in the absence of any commercial or financial relationships that could be construed as a potential conflict of interest.

## Supplementary material

Suppl. Figure I *Pgc1*-associated genes and mitoNEET levels in different organs. **(A)** mRNA levels *Ckb, MacroD1, Fabp3, Idh2, and Klf2* in PVAT of *Pgc1*α KO and *Pgc1*β KO mice. The relative mRNA level of each gene was normalized by 18S, respectively. Data shown are mean ± SD. *n* = 5 mice/group. **(B)** Representative blot shows mitoNEET protein levels in PVAT, BAT, gWAT, skeletal muscle, heart and brain in 10-week old C57BL/6J mice which were housed at 22°C or 4°C for 24 h. Quantification of mitoNEET levels in each tissue, normalized by β-actin. mitoNEET level in each tissue at 22°C was set as 100%. Quantification for PVAT, BAT, and WAT was calculated from two independent blots, and muscle, heart and brain were from one blot. Data shown as mean ± S.E.M.

Suppl. Figure II Brown adipocyte-specific mitoNEET overexpression mice. **(A)** Representative blot showing mitoNEET protein levels in adipose tissues in 10-week old wild type and mitoNEET-Tg mice. **(B)** Representative H.E. staining showing morphology of subcutaneous and gonadal BAT in 10-week old wild type and mitoNEET-Tg mice. Magnification bar = 20 μm.

Suppl. Figure III Increased in thermogenesis-related genes in gWAT and PVAT of mitoNEET-Tg mice. **(A)** MitoNEET-Tg mice were housed at 16°C for 1-week. RT-PCR was used to determine the mRNA levels (relative to 18S) of thermogenesis-related genes in gonadal WAT. Data shown as mean ± SD. *n* = 6 mice/group. **(B)** MitoNEET-Tg mice from line #27 were housed at 16°C for 1-week. RT-PCR was used to determine the mRNA levels (relative to 18S) of thermogenesis-related genes in PVAT. Data shown as mean ± SD. *n* = 6 mice/group.
