# Supplementary figures and images for: MitoNEET in Perivascular Adipose Tissue Blunts Atherosclerosis under Mild Cold Condition in Mice
Source: Front Physiol. 2017 Dec 19;8:1032. doi: 10.3389/fphys.2017.01032 (PMC5742148; doi:10.3389/fphys.2017.01032)

**Suppl. Figure I. *Pgc1*-associated genes and mitoNEET levels in different organs**

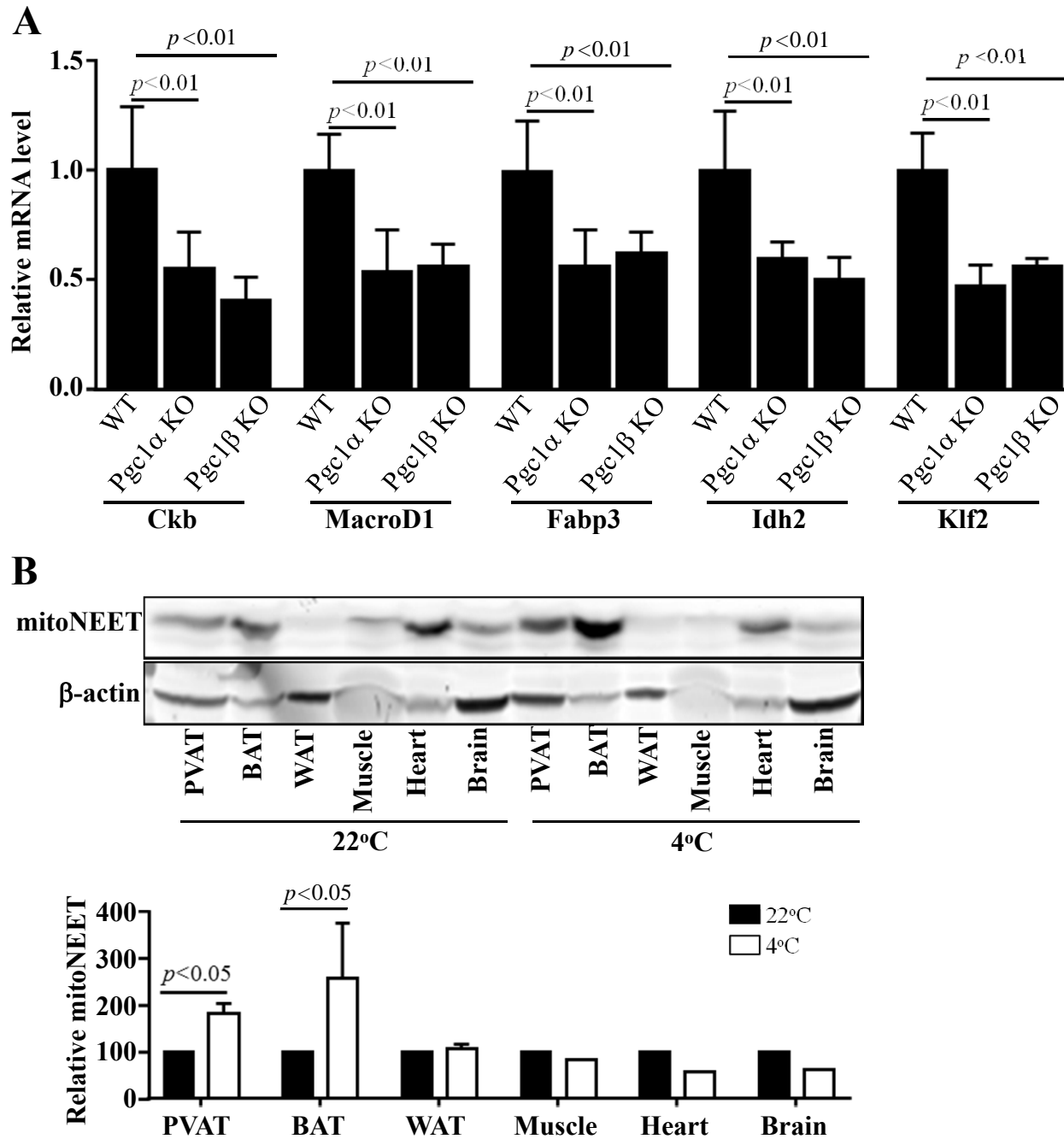

Supplement: Suppl. Figure I — Pgc1-associated genes and mitoNEET levels in different organs. (A) mRNA levels Ckb, MacroD1, Fabp3, Idh2, and Klf2 in PVAT of Pgc1α KO and Pgc1β KO mice. The relative mRNA level of each gene was normalized by 18S, respectively. Data shown are mean ± SD. n = 5 mice/group. (B) Representative blot shows mitoNEET protein levels in PVAT, BAT, gWAT, skeletal muscle, heart and brain in 10-week old C57BL/6J mice which were housed at 22°C or 4°C for 24 h. Quantification of mitoNEET levels in each tissue, normalized by β-actin. mitoNEET level in each tissue at 22°C was set as 100%. Quantification for PVAT, BAT, and WAT was calculated from two independent blots, and muscle, heart and brain were from one blot. Data shown as mean ± S.E.M. [file Image1.pdf]

**Suppl. Figure II. brown adipocyte-specific mitoNEET overexpression mice**

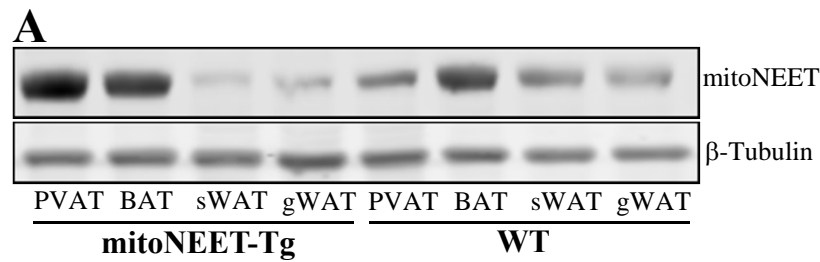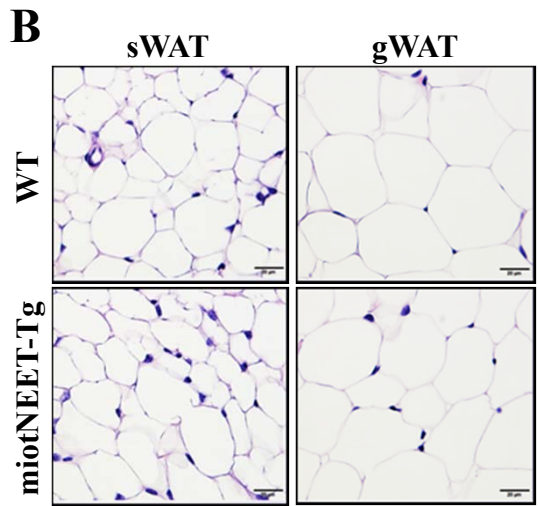

Supplement: Suppl. Figure II — Brown adipocyte-specific mitoNEET overexpression mice. (A) Representative blot showing mitoNEET protein levels in adipose tissues in 10-week old wild type and mitoNEET-Tg mice. (B) Representative H.E. staining showing morphology of subcutaneous and gonadal BAT in 10-week old wild type and mitoNEET-Tg mice. Magnification bar = 20 μm. [file Image2.pdf]

# Suppl. Figure III. Increased in thermogenesis-related genes in gWAT and PVAT of mitoNEET-Tg mice

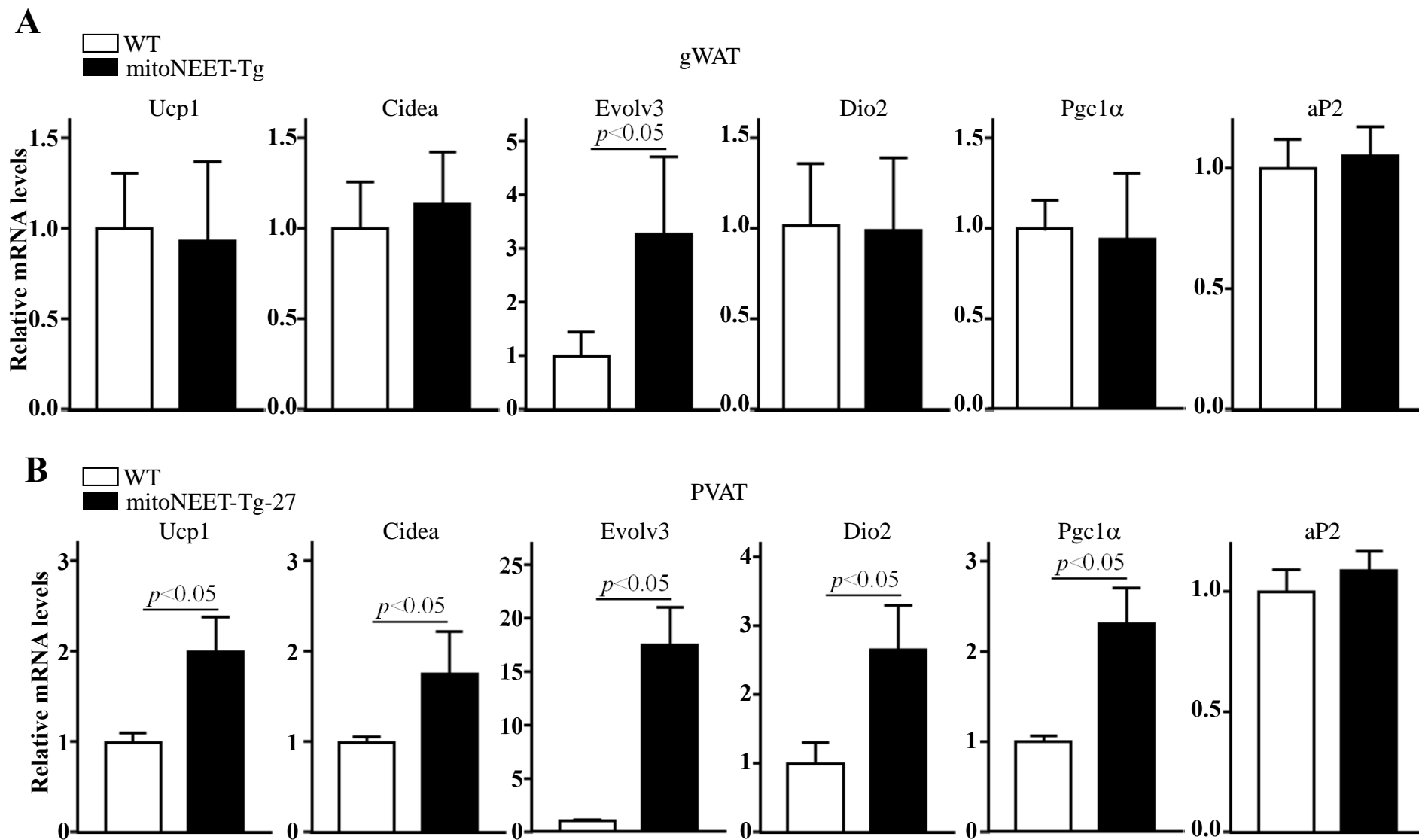

Supplement: Suppl. Figure III — Increased in thermogenesis-related genes in gWAT and PVAT of mitoNEET-Tg mice. (A) MitoNEET-Tg mice were housed at 16°C for 1-week. RT-PCR was used to determine the mRNA levels (relative to 18S) of thermogenesis-related genes in gonadal WAT. Data shown as mean ± SD. n = 6 mice/group. (B) MitoNEET-Tg mice from line #27 were housed at 16°C for 1-week. RT-PCR was used to determine the mRNA levels (relative to 18S) of thermogenesis-related genes in PVAT. Data shown as mean ± SD. n = 6 mice/group. [file Image3.pdf]
